# Identification and Characterization of vB_PreP_EPr2, a Lytic Bacteriophage of Pan-Drug Resistant *Providencia rettgeri*

**DOI:** 10.3390/v14040708

**Published:** 2022-03-29

**Authors:** Jaime L. Mencke, Yunxiu He, Andrey A. Filippov, Mikeljon P. Nikolich, Ashton T. Belew, Derrick E. Fouts, Patrick T. McGann, Brett E. Swierczewski, Derese Getnet, Damon W. Ellison, Katie R. Margulieux

**Affiliations:** 1 Wound Infections Department, Bacterial Diseases Branch, Walter Reed Army Institute of Research, Silver Spring, MD 20910, USA; jaime.mencke@usuhs.edu (J.L.M.); yunxiu.he.ctr@mail.mil (Y.H.); andrey.a.filippov.ctr@mail.mil (A.A.F.); mikeljon.p.nikolich.civ@mail.mil (M.P.N.); abelew@umd.edu (A.T.B.); derese.getnet.mil@mail.mil (D.G.); 2 F. Edward Hébert School of Medicine, Uniformed Services University of the Health Sciences, Bethesda, MD 20814, USA; 3 J. Craig Venter Institute, Rockville, MD 20850, USA; dfouts@jcvi.org; 4 Multidrug-Resistant Organism Repository and Surveillance Network (MRSN), Bacterial Diseases Branch, Walter Reed Army Institute of Research, Silver Spring, MD 20910, USA; patrick.t.mcgann4.civ@mail.mil; 5 Bacterial Diseases Branch, Walter Reed Army Institute of Research, Silver Spring, MD 20910, USA; brett.e.swierczewski.mil@mail.mil

**Keywords:** *Providencia rettgeri*, pan-drug resistance, bacteriophage, podophage, *Autographiviridae*, *Studiervirinae*, *Kayfunavirus*

## Abstract

*Providencia rettgeri* is an emerging opportunistic Gram-negative pathogen with reports of increasing antibiotic resistance. Pan-drug resistant (PDR) *P. rettgeri* infections are a growing concern, demonstrating a need for the development of alternative treatment options which is fueling a renewed interest in bacteriophage (phage) therapy. Here, we identify and characterize phage vB_PreP_EPr2 (EPr2) with lytic activity against PDR *P. rettgeri* MRSN 845308, a clinical isolate that carries multiple antibiotic resistance genes. EPr2 was isolated from an environmental water sample and belongs to the family *Autographiviridae*, subfamily *Studiervirinae* and genus *Kayfunavirus*, with a genome size of 41,261 base pairs. Additional phenotypic characterization showed an optimal MOI of 1 and a burst size of 12.3 ± 3.4 PFU per bacterium. EPr2 was determined to have a narrow host range against a panel of clinical *P. rettgeri* strains. Despite this fact, EPr2 is a promising lytic phage with potential for use as an alternative therapeutic for treatment of PDR *P. rettgeri* infections.

## 1. Introduction

*Providencia rettgeri* is a Gram-negative bacterial pathogen from the *Enterobacteriaceae* family and is an emerging cause of nosocomial infections [1,2]. Infections caused by *P. rettgeri* are typically linked to catheter placements and travelers’ diarrhea, but cellulitis, sepsis, and meningitis have also been reported, predominantly in immunocompromised individuals [2,3,4]. *P. rettgeri* is intrinsically resistant to the antibiotics colistin and tigecycline and has the ability to acquire additional drug resistance against antibiotic classes commonly used to treat infections [5]. Several studies have identified *P. rettgeri* strains that produce extended spectrum β-lactamases (ESBLs) and carbapenemases [6,7,8,9,10]. Increasing incidence of multidrug-resistant (MDR), extensively drug-resistant (XDR) and pan-drug resistant (PDR) *P. rettgeri* has been reported, severely limiting available treatment options in the clinical setting [7,8,9].

Bacteriophage (phage) therapy is an alternative treatment option being developed against bacterial infections, including those caused by drug-resistant strains. Phage mechanisms of action are distinct from those of antibiotics, and phages have been shown to target and kill MDR strains of both Gram-negative and Gram-positive bacterial species [11,12,13]. Recent publications have also demonstrated the potential benefit of phage-induced resensitization of MDR bacterial strains to antibiotics [14,15,16,17]. Additionally, the specificity of phages allows targeted treatment of bacterial infections without compromising patient’s normal microflora [12]. There are a limited number of lytic phages isolated and characterized with activity against *P. rettgeri* strains [18,19]. Identification of additional phages that target *P. rettgeri* will provide the opportunity to develop robust phage therapeutics against this emerging pathogen.

PDR *P. rettgeri* MRSN 845308 was isolated from a patient receiving care for COVID-19 in Texas, USA in December 2020 [9]. The patient’s symptoms did not improve with antibiotic therapy (vancomycin and cefepime), so the strain underwent further characterization by the Multidrug-Resistant Organism Repository and Surveillance Network (MRSN) at the Walter Reed Army Institute of Research (WRAIR). *P. rettgeri* MRSN 845308 was found to be non-susceptible to all available antibiotics and whole genome sequencing analysis showed that the strain possessed multiple antibiotic resistance genes, including *bla*_NDM-1_, *bla*_PER-1_, and *rmtB2* [9]. In this study, we report the isolation and characterization of phage vB_PreP_EPr2 (EPr2) from an environmental water sample with lytic activity against *P. rettgeri* MRSN 845308.

## 2. Materials and Methods

### 2.1. Bacterial Strains

Bacterial strains used in this study are listed in Table 1. *P. rettgeri* were obtained from the MRSN and the American Type Culture Collection (ATCC, Manassas, VA, USA). Bacteria were grown in Heart Infusion Broth (HIB) with shaking (200 rpm) or on HIB agar plates at 37 °C overnight.

### 2.2. Phage Isolation and Propagation

The phage EPr2 was isolated from environmental water collected from Rock Creek in Bethesda, MD, USA. The environmental water was filtered using sterile 0.22 µm filters (MilliporeSigma, Bollington, MA, USA) prior to phage enrichment. To enrich for *P. rettgeri* phages, 5 × HIB was mixed with the filter-sterilized environmental water sample at a 1:5 ratio and overnight broth culture of *P. rettgeri* MRSN 845308 was added. The enrichment mixture was incubated overnight at 37 °C with shaking at 200 rpm, after which the supernatant was sterilized using a 0.22 µm filter. The resulting lysate was assessed for phage activity against *P. rettgeri* MRSN 845308 through plating on double-layer HIB agar plates [20]. Phage purification was performed by three rounds of sequential single plaque isolation until uniform plaque morphology was achieved. Plaque morphology was analyzed using ImageJ software v. 1.53 (National Institutes of Health, Bethesda, MD, USA).

High titer phage lysates were obtained by liquid culture propagation of the phage in HIB supplemented with 0.5 mM CaCl_2_, 2 mM MgCl_2_, and 0.1% glucose. Phages were harvested after supernatant filtration with a 0.22 µm filter and centrifugation and stored in SM buffer (50 mM Tris-HCl, pH 7.5, 99 mM NaCl, 8 mM MgSO_4_, 0.01% gelatin) (Teknova, Hollister, CA, USA) at 4 °C protected from light. Plaque-forming units (PFUs) per ml were determined through 10-fold dilution in SM buffer and plating on double-layer agar plates.

### 2.3. Host Range Testing

The host range was determined against the *P. rettgeri* strains listed in Table 1 as described previously using a micro-spot dilution assay on square grid double-layer agar plates [21]. Lytic activity was determined positive if plaque formation was observed after overnight plate incubation at 37 °C. Host range testing was performed in two separate replicates with representative results reported.

### 2.4. Transmission Electron Microscopy

EPr2 was prepared for transmission electron microscopy (TEM) imaging as previously described [22]. Briefly, phages were washed twice with 0.1% ammonium acetate using high-speed centrifugation. Prepared phage particles were stained with 2% uranyl acetate for 1 min after being deposited on 300 mesh carbon-coated copper grids (Electron Microscopy Sciences, Hatfield, PA, USA). Samples were examined in a JEOL JEM-1400 electron microscope at 80 kv. Image analysis was conducted using Image J software v. 1.53 (National Institutes of Health, Bethesda, MD, USA).

### 2.5. Bacterial Strains Lytic Properties Assay

The dynamics of EPr2 lysis against *P. rettgeri* MRSN 845308 was determined as previously described [23]. Briefly, phage were mixed with a mid-log phase bacterial culture (OD_600_ = 0.5) at a multiplicity of infection (MOI) of 1, 0.1, 0.01, 0.001, and 0. The mixed culture was incubated at 37 °C with shaking at 200 rpm for the duration of the experiment. OD_600_ readings were taken every 10–20 min for 3 h. Experiments were repeated independently three times and analyzed using GraphPad Prism v. 9 (GraphPad Software, San Diego, CA, USA).

### 2.6. Determination of Optimal Multiplicity of Infection

The optimal MOI of EPr2 was determined as previously described [24]. Briefly, phage particles at various MOIs were added to mid-log phase bacterial culture (OD_600_ = 0.5) and incubated for 4 h at 37 °C with shaking at 200 rpm. The samples were centrifuged for 10 min and the supernatant was filtered using a 0.22 µm filter. Phage titer for each sample was determined by plating on double-layer agar plates. Experiments were repeated independently three times and analyzed using GraphPad Prism v. 9 (GraphPad Software, San Diego, CA, USA).

### 2.7. One Step Growth Curve

One-step growth curve experiments were performed as previously described [25]. Briefly, 1 mL of 5 × 10^6^ PFU/mL EPr2 stock was mixed with 1 mL of mid-log phase bacterial culture in HIB and allowed to adsorb for 5 min at 37 °C. The mixture was then diluted with fresh HIB and incubated at 37 °C for the duration of the experiment. Samples were taken every 5 min and plated using the double-layer agar plate method to determine phage titer. Experiments were repeated independently three times and analyzed using GraphPad Prism v. 9 (GraphPad Software, San Diego, CA, USA).

### 2.8. DNA Extraction and Whole Genome Sequencing

DNA extraction was performed using a modified QIAamp DNA Mini Kit (Qiagen, Germantown, MD, USA) protocol as described previously [26]. Briefly, a high-titer phage sample was treated with 2.5 U/mL DNase and 0.7 mg/mL RNase for 1.5 h at 37 °C, after which 20 mM EDTA was added to the sample. Next, the sample was treated with 3 µL of proteinase K and incubated for 1.5 h at 56 °C. An equal volume of Buffer AL was added to the treated phage lysate and QIAamp DNA Mini Kit instructions were followed to complete the DNA extraction.

Whole genome sequencing libraries were constructed from extracted EPr2 DNA using the KAPA HyperPlus Library preparation kit (Roche Diagnostics, Indianapolis, IN, USA). The prepared library was sequenced using MiSeq Reagent Kit v3 (600 cycle; 2 × 300 bp) (Illumina, San Diego, CA, USA). Whole genome sequencing analysis and phylogenetic analysis was performed as described in Supplementary Material Document S1. Briefly, paired end sequences were evaluated for quality then used for assembly with Unicycler [27]. The phage genome termini were determined using PhageTerm [28], and protein coding sequences (CDSs) were predicted and annotated using Prodigal [29]. The sequencing data is available in GenBank (accession number OM256482).

## 3. Results

### 3.1. Morphological Characterization of EPr2

**Figure 1:**
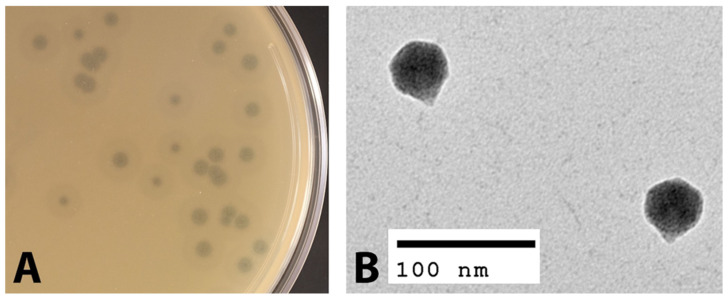
Morphological characterization of phage EPr2. (**A**) EPr2 plaque morphology on *P. rettgeri* MRSN 845308 and (**B**) TEM image.

The phage EPr2 was isolated from an environmental water sample from Rock Creek in Bethesda, Maryland, USA against the host strain *P. rettgeri* MRSN 845308. EPr2 forms large plaques of 2.4 ± 0.6 mm diameter with a halo that further extends 2.7 ± 0.3 mm from the edge of the plaque (Figure 1A). The phage particle morphology was observed using transmission electron microscopy (TEM). The TEM images showed that phage EPr2 has an icosahedral structure head with an approximate diameter of 43.7 ± 2.2 nm with a short non-contractile tail (Figure 1B). Based on these characteristics and in accordance with the International Committee on Taxonomy of Viruses (ICTV), EPr2 is morphologically classified as a podophage.

### 3.2. Host Range of EPr2

The host range of EPr2 was evaluated against 14 strains of *P. rettgeri*, 13 of which were clinical isolates collected by the MRSN from 2012–2021, and one strain was acquired from ATCC (Table 1). The results indicated that EPr2 has a narrow host range against the strains tested, only exhibiting lysis against three PDR isolates from the same patient within a short time period, including the initial host strain (Table 1). EPr2 was not active against other *P. rettgeri* strains collected from different sources.

### 3.3. Lytic Properties of EPr2

**Figure 2:**
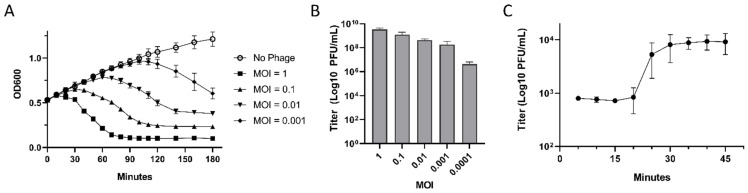
Lytic properties of phage EPr2 (**A**) EPr2 lysis dynamics against *P. rettgeri* MRSN 845308, (**B**) optimal MOI, and (**C**) one-step growth curve.

The dynamics of host strain lysis were measured at various MOIs of EPr2. The most rapid and robust lytic effect was demonstrated with an MOI of 1 starting after 30 min incubation and reaching full lysis at 90 min. Reduced MOIs resulted in increasing delay in and completeness of observed lysis (Figure 2A).

To determine optimal MOI of EPr2, mid log-phase cultures of *P. rettgeri* MRSN 845308 were incubated with phage MOIs of 0.0001 to 1. The highest phage titer achieved was 3.5 × 10^9^ PFU/mL with a starting MOI of 1 (Figure 2B). Lower starting MOIs resulted in decreasing total phage titer.

A one-step growth curve of EPr2 was also performed. The latent phase was estimated to be 20 min, followed by a rise phase of 10 min, and a plateau phase reached 30 min after initial infection (Figure 2C). The average burst size of EPr2 was estimated to be 12.3 ± 3.4 PFU per infected cell.

### 3.4. Genomic Analysis of EPr2

Phage EPr2 has a 41,261 base pair (bp) genome with a G + C content of 50.2% (GenBank accession no. OM256482). It has short 179 bp direct terminal repeats that were identified using PhageTerm [28]. Annotation of EPr2 resulted in the prediction of 69 CDSs. No antibiotic resistance genes, toxin genes, or other bacterial virulence genes were identified. BLASTn analysis against the NCBI non-redundant (NR) database assigned EPr2 to the family *Autographiviridae*, subfamily *Studiervirinae* and genus *Kayfunavirus*.

**Figure 3:**
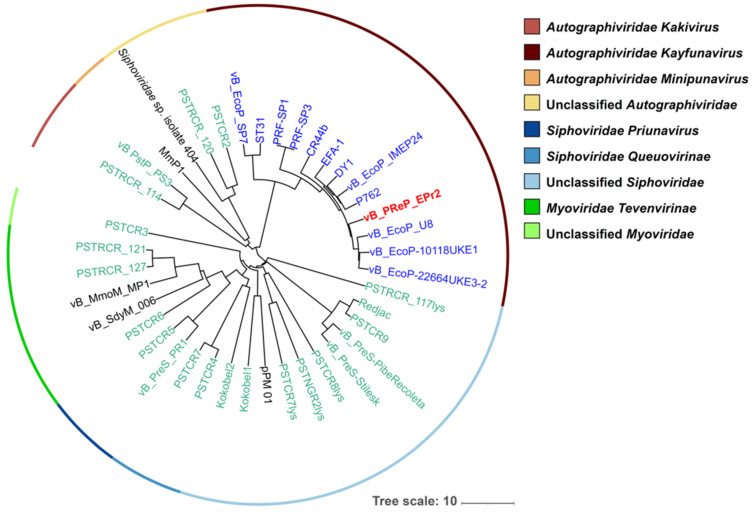
Phylogenetic tree of *Providencia* phage EPr2. A whole-genome average nucleotide distance tree was constructed for 40 total phage genomes (22 phages that infect *Providencia* species (green leaves), 5 phages related to known *Providencia* phages (black leaves), 12 with top BLASTn matches (blue leaves) and vB_PReP_EPr2 (bold red leaf) with MASH [30] (sketch size of s = 5000, k-mer size of k = 12 and GGRaSP [31] (see Supplementary Materials and Methods). Color strips denote taxonomic assignments (see key). The scale bar represents percent average nucleotide divergence.

The BLASTn results showed that EPr2 does not share high similarity with any known *Providencia* sp. phages. The most closely related phages to EPr2 targeted diverse bacterial host species, predominantly *Escherichia*, *Salmonella*, and *Citrobacter*. A phylogenetic tree was constructed with EPr2, 12 phages included in the top BLASTn results, 22 *Providencia* sp. phages, and 5 reference phages related to *Providencia* phages (Figure 3) [19]. Phage EPr2 was shown to share the most similarity with *Escherichia coli* phages through the phylogenetic analysis.

## 4. Discussion

*P. rettgeri* is an important emerging pathogen associated with drug-resistant hospital-associated infections. Several reports have identified *P. rettgeri* strains that harbor ESBL and carbapenemase genes, resulting in difficult to treat infections in a clinical setting, including the recent report of PDR *P. rettgeri* MRSN 845308 isolated from a patient being treated for COVID-19 [2,9]. Phage therapy offers a potential alternative treatment method for these MDR, XDR, and PDR strains [11,12].

In this study, we report the phenotypic and genomic characteristics of phage EPr2 with lytic activity against *P. rettgeri* MRSN 845308. Morphologically, EPr2 has an icosahedral head and short, non-contractile tail consistent with classic podovirus morphology. EPr2 forms large plaques with a halo, indicating production of a polysaccharide depolymerase and potential antibiofilm activity [32]. It has an optimal MOI of 1 and a relatively low burst size of 12.3 ± 3.4 PFU per bacterium. EPr2 belongs to the family *Autographiviridae*, subfamily *Studiervirinae* and genus *Kayfunavirus*, which include phages with short, non-contractile tails. To our knowledge, no other *Autographiviridae Kayfunavirus* phages that target *Providencia* sp. have been reported to date. Genomic analysis showed a 41,261 bp genome that appears to not carry any genes that would disqualify EPr2 from therapeutic use. The *P. rettgeri* strains tested are representative isolates collected from US military treatment facilities from 2012–2021. EPr2 has a narrow host range against this set, with lytic activity specific to the original PDR strains isolated within a short time period from the same patient. Interestingly, genome comparisons using NCBI BLASTn and phylogenetic analysis showed that EPr2 is the most similar to *Autographiviridae Kayfunavirus* phages that target *E. coli* and other *Enterobacteriaceae*, highlighting the lack of similar published *P. rettgeri* phages and an opportunity to further study potential cross-species lytic activity of EPr2.

Ideally, multiple phages would be used together in a cocktail with activity against a *P. rettgeri* infection to improve host range and minimize the opportunity for resistance to arise during therapy. However, there are currently limited *P. rettgeri* phages reported in the literature that may be suitable for therapeutic development. Oliveira et al. reported phage vB_PreS_PR1, a *Siphoviridae* phage with a plaque size of under 0.1 mm and broad host range activity against MDR *P. rettgeri* clinical isolates [18]. A study by Rakov et al. identified 12 phages with lytic activity against *Providencia stuartii* and *P. rettgeri* as part of the Israeli Phage Bank (IPB), including activity against biofilms [19]. The phages in that study represented *Siphoviridae*, *Myoviridae*, and *Autographiviridae* phylogenetic families and 39/41 *Providencia* sp. strains showed susceptibility to at least one phage [19]. In comparison, EPr2 forms large plaques with a halo, belongs to the *Autographiviridae* family, and has a narrow host range. This indicates a broad diversity of *P. rettgeri* phages and suggests that discovery of additional phages is required for the development of a broad-coverage therapeutic phage cocktail.

In summary, we report the isolation and characterization of EPr2, a lytic phage with activity against the PDR clinical isolate *P. rettgeri* MRSN 845308. This study provides evidence that phage treatment is a viable alternative therapeutic option for PDR *P. rettgeri* infections. Importantly, EPr2 was discovered and isolated in one week from a single environmental source, indicating phages that target clinical *P. rettgeri* strains can be identified rapidly and on demand. EPr2 demonstrates desirable characteristics for a therapeutic phage, including strong lytic activity against a PDR strain of interest and no genes indicative of a lysogenic lifestyle, drug resistance, or virulence. It is important to continue phage hunting for *Providencia* sp. phages as infections rise and drug resistance profiles continue to evolve, rendering most or all available antibiotics ineffective.

## Figures and Tables

**Table 1:** Host Range of EPr2.

*P. rettgeri* Strain ID	Collection Date	Isolate Source	EPr2 Sensitivity ^3^
MRSN 12965	4 December 2012	Surveillance	−
MRSN 21225	3 March 2014	Respiratory	−
MRSN 26563	13 October 2014	Urine	−
MRSN 31245	23 July 2015	Stool	−
MRSN 32788	10 August 2015	Stool	−
MRSN 33475	24 August 2015	Wound	−
MRSN 386496	22 April 2016	Urine	−
MRSN 768611	26 March 2020	Urine	−
MRSN 845308 ^1,2^	1 November 2020	Blood	+
MRSN 849144 ^2^	6 November 2020	Surveillance	+
MRSN 849312 ^2^	9 November 2020	Blood	+
MRSN 877360	25 December 2020	Urine	−
MRSN 883526	24 February 2021	Surveillance	−
ATCC 29944	N/A ^4^	N/A	−

^1^ EPr2 host strain. ^2^ Strains isolated from same patient. ^3^ “−“ = no sensitivity, “+” = sensitivity. ^4^ N/A = not available.

## Data Availability

The whole genome sequence of vB_PreP_EPr2 has been deposited in GenBank under the accession number OM256482.

## Supplementary Materials

The following supporting information can be downloaded at: https://www.mdpi.com/article/10.3390/v14040708/s1, Supplemental Materials and Methods Document S1. References [33,34,35,36,37,38,39,40,41,42,43,44,45,46,47,48,49,50,51,52,53,54,55,56,57,58,59,60,61,62,63] are cited in the supplementary materials.
