# Supplementary material for: Identification and Characterization of vB_PreP_EPr2, a Lytic Bacteriophage of Pan-Drug Resistant Providencia rettgeri
Source: Viruses. 2022 Mar 29;14(4):708. doi: 10.3390/v14040708 (PMC9026810; doi:10.3390/v14040708)
Supplement: Supplementary file 1 [file viruses-14-00708-s001.zip › viruses-1585128-supplementary.pdf]

Supplemental Material and Methods

Bioinformatic Analysis of EPr2

Paired end sequences (108, 202 reads total) were evaluated for quality with FastQC (https://www.bioinformatics.babraham.ac.uk/projects/fastqc/) and trimmed with Trimmomatic [33]. A total of 95,613 trimmed reads passed and were corrected with RACER [34]. Kraken2 classification of the resulting reads was performed using the default bacterial database [35] to identify a bacterial host sequence from which contaminating reads would be derived and removed. EPr2 has a previously sequenced host strain, *P. rettgeri* MRSN 845308, which was selected and indexed instead. The reads were aligned against the manually chosen host strain with Hisat2 [36] with options to separately save the aligned (119 reads) and unaligned reads (95,494 reads). The unaligned reads were chosen as the input for all following tasks. These host-filtered reads were passed again to Kraken2 and run using the viral database supplemented with the set of reference genomes provided by the International Committee on Taxonomy of Viruses (ICTV) [37]. These filtered/cleaned/trimmed reads were passed to Unicycler [27] and SPAdes [38] in order to create the initial assembly.

The assembly resulted in one contig and coverage was assessed. The assembled contig was passed to phastaf (https://github.com/tseemann/phastaf) for an initial taxonomy query of the assembly. A further taxonomic query was performed by aligning the catalog of all ICTV reference genomes against the assemblies with Fasta36 [39]. An initial set of putative CDS sequences was produced with Prodigal [29], counted by strand, and the majority strand was chosen as the Watson strand. The phage terminus was determined with PhageTerm [28] using the Unicycler assembly and filtered reads; when possible the resulting genome was reoriented

to put the DTR at the beginning. If this did not succeed, Fasta36 was used with a database of phage terminase genes taken from the Manual Annotation Studio (MAS) program [40] to reorient the assembly.

The first set of annotations was determined using Prokka [41] followed by additional CDS searches using a phage-trained Prodigal instance, an untrained (and/or trained against the same phage) run of Glimmer3 [42], and the set of predictions generated by Phanotate [43]. The Phanotate CDS predictions were chosen as the primary set and supplemented by the Prodigal and Glimmer results; thus when the start/stop coordinates matched those from Prodigal, the Shine-Dalgarno, etc. annotations were extracted and added. These CDS predictions were merged with the initial Prokka data and the resulting GenBank and/or fasta files were used as input for a series of downstream searches and analyses. The k-mer content of each assembly was queried via Jellyfish [44] with k spanning a putatively useful range of values (9, 11, 13, 15, 17). The Prokka-derived tRNA detections were supplemented with an explicit tmRNA search via ARAGORN [45] followed by a permissive search with tRNAscan-SE [46] which may optionally be added to the final assembly. The CDS nucleotide sequences were passed to Trinotate [47] with a template file directing it to search additionally against a blast database of all ICTV reference assemblies and terminase genes. Resistance genes were sought via ABRicate (https://github.com/tseemann/abricate) with the full set of associated databases (AMRFinderPlus [48], CARD [49], ResFinder [50], ARG-ANNOT [51], VFDB [52], PlasmidFinder [53], EcOH [54], and MEGARes [55]; this set was supplemented with mVirDB [56] (using a copy provided by the internet archive and filtered to separate the nucleotide and protein sequences:

https://web.archive.org/web/20161123071504/http://mvirdb.llnl.gov/annotation/bin/downloadTarFiles.pl?file=/www/annotation/db/virulence/blastMvirDB.tar.gz) and DBETH [57]. The amino acid sequences were passed to InterProScan [58] with all methods enabled. The above tasks were performed and the resulting annotations and putative features were merged together via a modified version of portions of Prokka via CYOA (http://github.com/abelew/cyoa).

Finally, the resulting assemblies were passed to CGView [59] for visualization. The assemblies were passed to a 201 nucleotide rolling window scan using RNAfold [60], all restriction sites were collected via BioPerl's Bio::Restriction::Analysis, and metadata collected via hpgltools (https://github.com/elsayed-lab/hpgltools).

A phage whole genome phylogeny was generated from an ANI-based distance matrix calculated with the MASH program [30] as described previously [61]. Briefly, a sketch file was created from the described *Providencia* phage genome isolated and sequenced in this study plus 39 obtained from GenBank (22 *Providencia* phages, 5 related phages related to known *Providencia* phages [19] and 12 with BLASTn matches to vB_PReP_EPr2), with 5000 12mers generated per genome (i.e., mash sketch -s 5000 -K 12). The sketch file was then compared to all phage genome sequences to generate the ANI distance matrix with the MASH distance command using default settings. The GGRaSP [31] R-package was used to calculate the UPGMA phylogeny from the ANI distance matrix, after redundant phage genomes (genomes ANI > 99.985) were removed using the GGRaSP R package with a user defined cutoff of 0.015 (i.e., GGRaSP -threshold=0.015)). The resulting dendrogram was translated into newick format within GGRaSP using the APE R package [62], loaded into the iTOL tree viewer [63], and annotated with taxonomic information.

References

19. Rakov, C.; Ben Porat, S.; Alkalay-Oren, S.; Yerushalmy, O.; Abdalrhman, M.; Gronovich, N.; Huang, L.; Pride, D.; Coppenhagen-Glazer, S.; Nir-Paz, R.; Hazan, R., Targeting biofilm of MDR *Providencia stuartii* by phages using a catheter model. *Antibiotics* **2021,** 10, (4), 375.
27. Wick, R. R.; Judd, L. M.; Gorrie, C. L.; Holt, K. E., Unicycler: Resolving bacterial genome assemblies from short and long sequencing reads. *PLoS Comput Biol* **2017,** 13, (6), e1005595.
28. Garneau, J. R.; Depardieu, F.; Fortier, L. C.; Bikard, D.; Monot, M., PhageTerm: a tool for fast and accurate determination of phage termini and packaging mechanism using next-generation sequencing data. *Sci Rep* **2017,** 7, (1), 8292.
29. Hyatt, D.; Chen, G. L.; Locascio, P. F.; Land, M. L.; Larimer, F. W.; Hauser, L. J., Prodigal: prokaryotic gene recognition and translation initiation site identification. *BMC Bioinformatics* **2010,** 11, 119.
30. Ondov, B. D.; Treangen, T. J.; Melsted, P.; Mallonee, A. B.; Bergman, N. H.; Koren, S.; Phillippy, A. M., Mash: fast genome and metagenome distance estimation using MinHash. *Genome Biol* **2016,** 17, (1), 132.
31. Clarke, T. H.; Brinkac, L. M.; Sutton, G.; Fouts, D. E., GGRaSP: a R-package for selecting representative genomes using Gaussian mixture models. *Bioinformatics (Oxford, England)* **2018,** 34, (17), 3032-3034.
33. Bolger, A. M.; Lohse, M.; Usadel, B., Trimmomatic: a flexible trimmer for Illumina sequence data. *Bioinformatics (Oxford, England)* **2014,** 30, (15), 2114-20.
34. Ilie, L.; Molnar, M., RACER: Rapid and accurate correction of errors in reads. *Bioinformatics (Oxford, England)* **2013,** 29, (19), 2490-3.
35. Wood, D. E.; Lu, J.; Langmead, B., Improved metagenomic analysis with Kraken 2. *Genome Biol* **2019,** 20, (1), 257.
36. Kim, D.; Paggi, J. M.; Park, C.; Bennett, C.; Salzberg, S. L., Graph-based genome alignment and genotyping with HISAT2 and HISAT-genotype. *Nat Biotechnol* **2019,** 37, (8), 907-915.
37. Lefkowitz, E. J.; Dempsey, D. M.; Hendrickson, R. C.; Orton, R. J.; Siddell, S. G.; Smith, D. B., Virus taxonomy: the database of the International Committee on Taxonomy of Viruses (ICTV). *Nucleic Acids Res* **2018,** 46, (D1), D708-D717.
38. Bankevich, A.; Nurk, S.; Antipov, D.; Gurevich, A. A.; Dvorkin, M.; Kulikov, A. S.; Lesin, V. M.; Nikolenko, S. I.; Pham, S.; Prjibelski, A. D.; Pyshkin, A. V.; Sirotkin, A. V.; Vyahhi, N.; Tesler, G.; Alekseyev, M. A.; Pevzner, P. A., SPAdes: a new genome assembly algorithm and its applications to single-cell sequencing. *J Comput Biol* **2012,** 19, (5), 455-77.
39. Pearson, W. R., Finding protein and nucleotide similarities with FASTA. *Curr Protoc Bioinformatics* **2016,** 53, 3.9.1-3.9.25.
40. Lueder, M. R.; Cer, R. Z.; Patrick, M.; Voegtly, L. J.; Long, K. A.; Rice, G. K.; Bishop-Lilly, K. A., Manual Annotation Studio (MAS): a collaborative platform for manual functional annotation of viral and microbial genomes. *BMC Genomics* **2021,** 22, (1), 733.
41. Seemann, T., Prokka: rapid prokaryotic genome annotation. *Bioinformatics (Oxford, England)* **2014,** 30, (14), 2068-9.
42. Delcher, A. L.; Bratke, K. A.; Powers, E. C.; Salzberg, S. L., Identifying bacterial genes and endosymbiont DNA with Glimmer. *Bioinformatics (Oxford, England)* **2007,** 23, (6), 673-9.
43. McNair, K.; Zhou, C.; Dinsdale, E. A.; Souza, B.; Edwards, R. A., PHANOTATE: a novel approach to gene identification in phage genomes. *Bioinformatics (Oxford, England)* **2019,** 35, (22), 4537-4542.
44. Marcais, G.; Kingsford, C., A fast, lock-free approach for efficient parallel counting of occurrences of k-mers. *Bioinformatics (Oxford, England)* **2011,** 27, (6), 764-70.

45. Laslett, D.; Canback, B., ARAGORN, a program to detect tRNA genes and tmRNA genes in nucleotide sequences. *Nucleic Acids Res* **2004,** 32, (1), 11-6.
46. Chan, P. P.; Lowe, T. M., tRNAscan-SE: Searching for tRNA genes in genomic sequences. *Methods Mol Biol* **2019,** 1962, 1-14.
47. Bryant, D. M.; Johnson, K.; DiTommaso, T.; Tickle, T.; Couger, M. B.; Payzin-Dogru, D.; Lee, T. J.; Leigh, N. D.; Kuo, T. H.; Davis, F. G.; Bateman, J.; Bryant, S.; Guzikowski, A. R.; Tsai, S. L.; Coyne, S.; Ye, W. W.; Freeman, R. M., Jr.; Peshkin, L.; Tabin, C. J.; Regev, A.; Haas, B. J.; Whited, J. L., A tissue-mapped axolotl transcriptome enables identification of limb regeneration factors. *Cell Rep* **2017,** 18, (3), 762-776.
48. Feldgarden, M.; Brover, V.; Haft, D. H.; Prasad, A. B.; Slotta, D. J.; Tolstoy, I.; Tyson, G. H.; Zhao, S.; Hsu, C. H.; McDermott, P. F.; Tadesse, D. A.; Morales, C.; Simmons, M.; Tillman, G.; Wasilenko, J.; Folster, J. P.; Klimke, W., Validating the AMRFinder Tool and Resistance Gene Database by Using Antimicrobial Resistance Genotype-Phenotype Correlations in a Collection of Isolates. *Antimicrob Agents Chemother* **2019,** 63, (11).
49. Jia, B.; Raphenya, A. R.; Alcock, B.; Waglechner, N.; Guo, P.; Tsang, K. K.; Lago, B. A.; Dave, B. M.; Pereira, S.; Sharma, A. N.; Doshi, S.; Courtot, M.; Lo, R.; Williams, L. E.; Frye, J. G.; Elsayegh, T.; Sardar, D.; Westman, E. L.; Pawlowski, A. C.; Johnson, T. A.; Brinkman, F. S.; Wright, G. D.; McArthur, A. G., CARD 2017: expansion and model-centric curation of the comprehensive antibiotic resistance database. *Nucleic Acids Res* **2017,** 45, (D1), D566-D573.
50. Zankari, E.; Hasman, H.; Cosentino, S.; Vestergaard, M.; Rasmussen, S.; Lund, O.; Aarestrup, F. M.; Larsen, M. V., Identification of acquired antimicrobial resistance genes. *J Antimicrob Chemother* **2012,** 67, (11), 2640-2644.
51. Gupta, S. K.; Padmanabhan, B. R.; Diene, S. M.; Lopez-Rojas, R.; Kempf, M.; Landraud, L.; Rolain, J. M., ARG-ANNOT, a new bioinformatic tool to discover antibiotic resistance genes in bacterial genomes. *Antimicrob Agents Chemother* **2014,** 58, (1), 212-20.
52. Chen, L.; Zheng, D.; Liu, B.; Yang, J.; Jin, Q., VFDB 2016: hierarchical and refined dataset for big data analysis--10 years on. *Nucleic Acids Res* **2016,** 44, (D1), D694-7.
53. Carattoli, A.; Zankari, E.; Garcia-Fernandez, A.; Voldby Larsen, M.; Lund, O.; Villa, L.; Moller Aarestrup, F.; Hasman, H., *In silico* detection and typing of plasmids using PlasmidFinder and plasmid multilocus sequence typing. *Antimicrob Agents Chemother* **2014,** 58, (7), 3895-903.
54. Ingle, D. J.; Valcanis, M.; Kuzevski, A.; Tauschek, M.; Inouye, M.; Stinear, T. P.; Levine, M. M.; Robins-Browne, R. M.; Holt, K. E., *In silico* serotyping of E. coli from short read data identifies limited novel O-loci but extensive diversity of O:H serotype combinations within and between pathogenic lineages. *Microbial genomics* **2016,** 2, (7), e000064.
55. Doster, E.; Lakin, S. M.; Dean, C. J.; Wolfe, C.; Young, J. G.; Boucher, C.; Belk, K. E.; Noyes, N. R.; Morley, P. S., MEGARes 2.0: a database for classification of antimicrobial drug, biocide and metal resistance determinants in metagenomic sequence data. *Nucleic Acids Res* **2020,** 48, (D1), D561-D569.
56. Zhou, C. E.; Smith, J.; Lam, M.; Zemla, A.; Dyer, M. D.; Slezak, T., MvirDB--a microbial database of protein toxins, virulence factors and antibiotic resistance genes for bio-defence applications. *Nucleic Acids Res* **2007,** 35, (Database issue), D391-4.
57. Chakraborty A; Ghosh S; Chowdhary G; Maulik U; S, C., DBETH: a database of bacterial exotoxins for human. *Nucleic Acids Res* **2012,** 40, D615-20.
58. Jones, P.; Binns, D.; Chang, H. Y.; Fraser, M.; Li, W.; McAnulla, C.; McWilliam, H.; Maslen, J.; Mitchell, A.; Nuka, G.; Pesseat, S.; Quinn, A. F.; Sangrador-Vegas, A.; Scheremetjew, M.; Yong, S. Y.; Lopez, R.; Hunter, S., InterProScan 5: genome-scale protein function classification. *Bioinformatics (Oxford, England)* **2014,** 30, (9), 1236-40.

59. Stothard, P.; Wishart, D. S., Circular genome visualization and exploration using CGView. *Bioinformatics (Oxford, England)* **2005,** 21, (4), 537-9.
60. Lorenze, R.; Bernhart, S. H.; Z., S. C. H.; Tafer, H.; Flamm, C.; Stadler, P. F.; Hofacker, I. L., ViennaRNA Package 2.0. *Algorithms Mol Biol* **2011,** 6, 26.
61. Duan, Y.; Llorente, C.; Lang, S.; Brandl, K.; Chu, H.; Jiang, L.; White, R. C.; Clarke, T. H.; Nguyen, K.; Torralba, M.; Shao, Y.; Liu, J.; Hernandez-Morales, A.; Lessor, L.; Rahman, I. R.; Miyamoto, Y.; Ly, M.; Gao, B.; Sun, W.; Kiesel, R.; Hutmacher, F.; Lee, S.; Ventura-Cots, M.; Bosques-Padilla, F.; Verna, E. C.; Abraldes, J. G.; Brown, R. S., Jr.; Vargas, V.; Altamirano, J.; Caballeria, J.; Shawcross, D. L.; Ho, S. B.; Louvet, A.; Lucey, M. R.; Mathurin, P.; Garcia-Tsao, G.; Bataller, R.; Tu, X. M.; Eckmann, L.; van der Donk, W. A.; Young, R.; Lawley, T. D.; Starkel, P.; Pride, D.; Fouts, D. E.; Schnabl, B., Bacteriophage targeting of gut bacterium attenuates alcoholic liver disease. *Nature* **2019,** 575, (7783), 505-511.
62. Paradis, E.; Schliep, K., ape 5.0: an environment for modern phylogenetics and evolutionary analyses in R. *Bioinformatics (Oxford, England)* **2019,** 35, (3), 526-528.
63. Letunic, I.; Bork, P., Interactive tree of life (iTOL) v3: an online tool for the display and annotation of phylogenetic and other trees. *Nucleic Acids Res* **2016,** 44, (W1), W242-5.
